# Effects of Light Intensity on the Growth, Photosynthetic Characteristics, and Flavonoid Content of *Epimedium pseudowushanense* B.L.Guo

**DOI:** 10.3390/molecules21111475

**Published:** 2016-11-04

**Authors:** Junqian Pan, Baolin Guo

**Affiliations:** Institute of Medicinal Plant Development, Chinese Academy of Medical Sciences, Peking Union Medical College, Beijing 100193, China; panjunqian123@163.com

**Keywords:** *Epimedium pseudowushanense* B.L.Guo, light intensity growth, photosynthetic characteristics, flavonoid content, medicinal-ingredient yield

## Abstract

*Epimedium pseudowushanense* B.L.Guo is used in traditional medicine as an aphrodisiac and to strengthen muscles and bones. Several recent reports have shown that flavonoids from *Epimedium* also significantly affect the treatment of breast cancer, liver cancer, and leukemia. However, few studies have examined the medicinal-ingredient yield of *Epimedium*, a light-demanding shade herb, under different light intensities. To investigate the effects of light intensity on medicinal-ingredient yields, *Epimedium* was exposed to five levels of light intensity until harvest time. Leaf dry biomass under L4 was the highest among different light treatments. L4 was also associated with the highest net photosynthetic rate. Quantification of epimedin A, epimedin B, epimedin C, and icariin showed that L3 produced the highest amount of epimedin C, and that flavonoid content responded to light levels differently. Results indicated that L3 and L4 were the optimal light levels for medicinal-ingredient yield.

## 1. Introduction

*Epimedii Folium*, commonly called Yinyanghuo, is an important perennial shade herb in traditional Chinese medicine. *Epimedium* belongs to the Berberidaceae family and is widely used as an aphrodisiac and to strengthen muscles and bones. *Epimedium* leaves contain high amounts of flavonoid glycosides, such as epimedin A, epimedin B, epimedin C, and icariin [1,2]. Recent reports have shown that flavonoids from *Epimedium* significantly affect the treatment of breast cancer, liver cancer, and leukemia [3,4,5]. The recognition of the health benefits of *Epimedium* has increased its market demand. However, its resource recycling rate is low and environmentally dependent. Furthermore, its natural sources are endangered, further increasing prices. Commercial culture can address resource constraints of *Epimedium*. *E. pseudowushanense* B.L.Guo is an important resource of *Epimedii Folium* and is the first *Epimedium* species introduced and cultivated in Guizhou Province, China.

Light is an important environmental factor influencing plant growth, development, and secondary metabolism [6]. Under high light conditions, certain plant species, such as bayberry trees, do not grow because light irradiance decreases photosynthetic rates [7]. Therefore, all plants have their own optimal light intensity ranges for growth. Light intensity that is too high or too low impacts morphology, photosynthetic physiology, and secondary metabolite production. These characteristics are closely related to medicinal plant productivity. For example, Ma and others [8] reported that *Camptotheca acuminata* grown under 75% irradiance had significantly greater height, net photosynthetic rate (Pn), stomatal conductance (gs), total aboveground biomass, and chlorophyll fluorescence than plants grown under 100% (1500 ± 30 μmol·m^−2^·s^−1^), 50%, and 25% irradiance.

The function of different flavonoids varies according to the structure and specific flavonoids are involved for instance in limiting exogenous microbial growth, helping generate new fruits or leaves, providing light protection, and enhancing antioxidant defense [9,10]. Qualitative and quantitative composition of flavonoids varies under different environments [9]. Changes in light intensity may influence flavonoid content because the flavonoid hydroxyl groups on the A and B rings vary in number and position. Several studies have shown that high light irradiance promotes the biosynthesis of flavonoids, such as dihydroxy B-ring-substituted flavonoids (luteolin 7-*O*- and quercetin 3-*O*-glycosides) but does not influence the biosynthesis of monohydroxy B-ring-substituted flavonoids (pigenin 7-*O*- and kaempferol 3-*O*-glycosides) [11,12,13,14]. Pacheco and others [15] reported that *Piper aduncum* grown under 50% natural light irradiance had higher total flavonoid concentration than those grown under 100% natural irradiance. Based on these reports and the harvest time of *E. pseudowushanense*, the present study exposed *Epimedium* to five different light intensities for 60 days.

In the natural environment, *E. pseudowushanense* grows in woodlands, scrub, and forest edges. In commercial culture, *E. pseudowushanense* seedlings are usually shade-grown by a simulated wild cultivation method. However, our understanding of the photosynthesis and flavonoid content of *E. pseudowushanense* under shade management is limited. This study aims to determine the optimal light conditions for commercial *Epimedium* production. This study also aims to investigate flavonoid accumulation in *Epimedium* under different light intensities to improve its propagation and cultivation. The results of the present study may contribute to the scientific culture and management of *Epimedium*, as well as provide a reference for other *Epimedium* species. We anticipate that our results will improve our understanding of the changes in photosynthetic parameters and concentrations of secondary metabolites in *Epimedium* under different light treatments. Our results will provide a sound theoretical foundation for the standardized cultivation of this important medicinal plant.

## 2. Results

### 2.1. Effects of Light Intensity on Plant Growth

**Figure 1:**
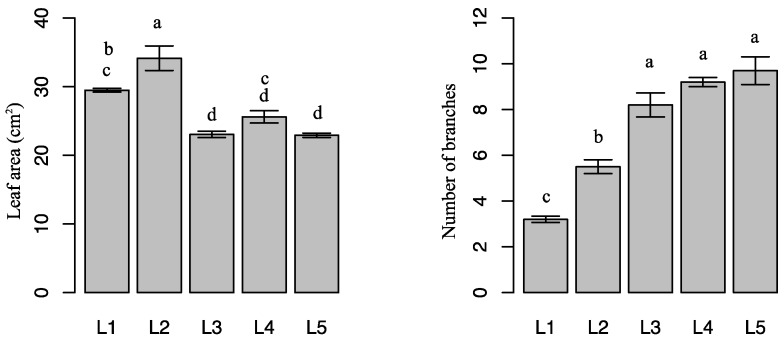

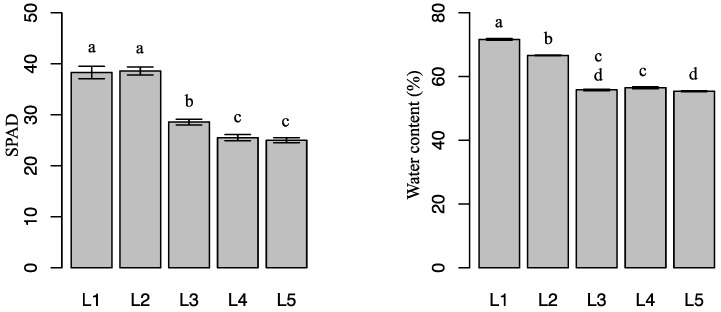
Morphological parameters of *E. pseudowushanense* under different light intensities. The data are expressed as mean ± SD. Different letters indicate significant differences between light intensity treatments (*p* < 0.05); *n* = 30.

Clear external differences were observed among plants grown under 60 days of different light intensities. Compared with high light intensities (L3, L4, and L5), leaf areas were higher under low light intensities (L1 and L2). L2 resulted in the highest leaf area. Light intensity had different effects on *E. pseudowushanense* growth: L3, L4, and L5 resulted in higher number of branches. On the contrary, L1 and L2 resulted in higher SPAD and water content. The number of branches under L3, L4, and L5 were 156.3% (*p* < 0.05), 187.5% (*p* < 0.05), and 203.1% (*p* < 0.05) higher, respectively, than of plants grown under L1. SPAD values under L1 and L2 were 53.1% (*p* < 0.05) and 54.3% (*p* < 0.05) higher, respectively, than in plants grown under L5. Water content responded similarly. The highest values were observed in plants under L1 and the lowest were in plants under L5 (Figure 1).

### 2.2. Effects of Light Intensity on Plant Photosynthetic Parameters

**Figure 2:**
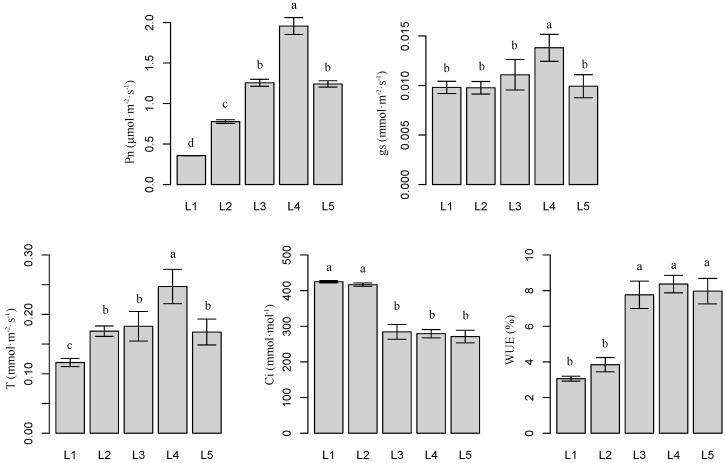
Net photosynthetic rate (Pn), stomatal conductance (gs), intercellular CO_2_ concentration (Ci), transpiration rate (T), and water use efficiency (WUE) of *E. pseudowushanense* leaves under different light intensities. The data are expressed as mean ± SD; *n* = 9. Different letters indicate significant differences between light intensity treatments (*p* < 0.05).

Figure 2 presents the effects of different light intensities on leaf photosynthetic parameters. Compared with L1, leaf net photosynthesis (Pn), transpiration rate (T), and water use efficiency (WUE) increased as light intensity increased, but not under L5. L4 resulted in the highest Pn, T, and WUE, whereas L1 resulted in the lowest measurements. Compared with L1, L4 increased Pn, T, and WUE by 457.9% (*p* < 0.05), 107.6% (*p* < 0.05), and 173.7% (*p* < 0.05), respectively. The highest gs values were observed under L4. No significant differences were observed between other treatments (*p* > 0.05). Compared with L1, intercellular CO_2_ concentration (Ci) decreased by 33.1% (*p* < 0.05), 34.3% (*p* < 0.05), and 36.2% (*p* < 0.05) under L3, L4, and L5, respectively.

### 2.3. Effects of Light Intensity on Chloroplast Ultrastructure

**Figure 3:**
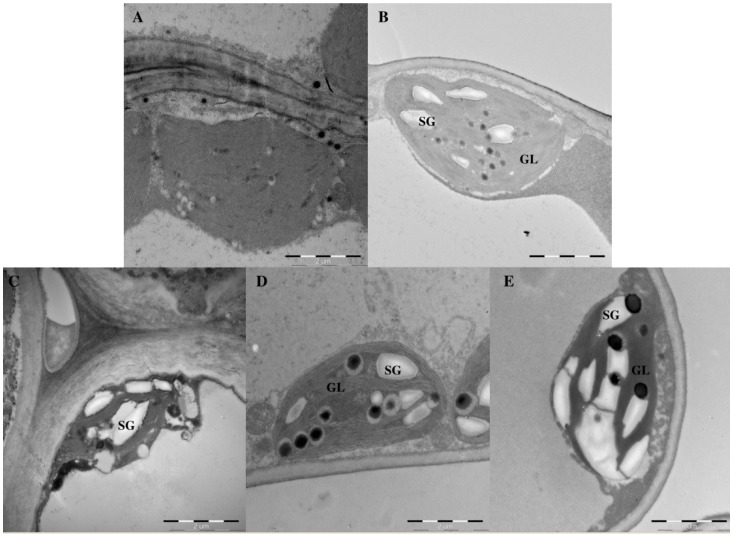
Chloroplast ultrastructure of *E. pseudowushanense* mesophyll cells under L1 (**A**); L2 (**B**); L3 (**C**); L4 (**D**); and L5 (**E**) treatment at 60 days. Abbreviation: SG, starch grains; GL, grana lamellae.

Light intensity markedly influenced the shape and number of starch grains and grana lamellae in *Epimedium* (Figure 3). L4 resulted in more grana lamellae than other treatments, with approximately 11 to 14 grana lamellae per grana. Plants under L5 had more starch grains per chloroplast. Starch grains seldom appeared under L1. Interestingly, plants under L1 and L2 had narrower chloroplasts than plants under L3, L4, and L5.

### 2.4. Effect of Light Intensity on Flavonoid Content in Epimedium

**Figure 4:**
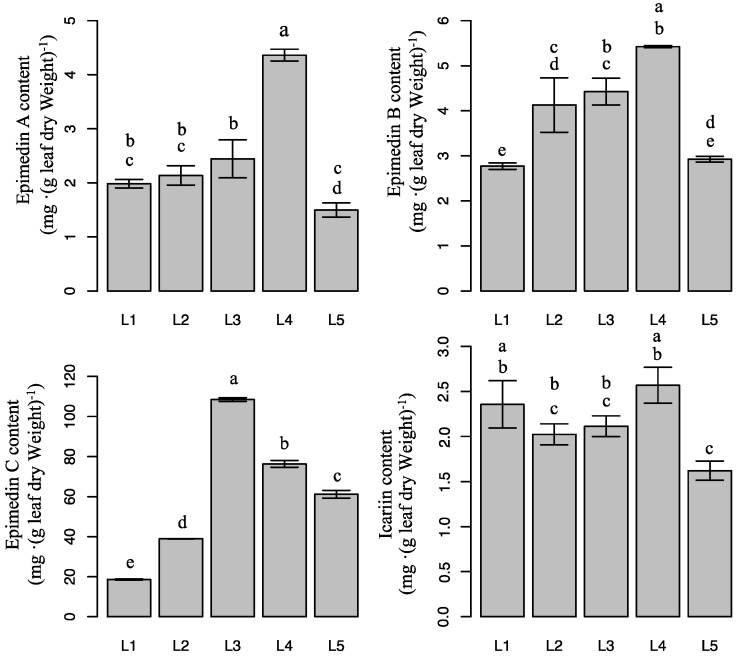
Epimedin A, epimedin B, epimedin C, and icariin content of *E. pseudowushanense* under different light intensities. The data are expressed as mean ± SD. Different letters indicate significant differences between light intensity treatments (*p* < 0.05); *n* = 30.

Table 1 shows that the method of determining the flavonoid glycosides is highly accurate. Figure 4 shows the changes in the contents of four different flavonoid glycosides in *E. pseudowushanense* under different light intensities. Epimedin A and epimedin B amounts showed similar changes. Epimedin A and epimedin B increased when light intensity increased from L1 to L4, whereas both decreased under L5. The highest epimedin A and epimedin B contents were observed under L4. Epimedin A and epimedin B contents were 191.4% (*p* < 0.05) and 95.8% (*p* < 0.05) higher, respectively, than under L1. Furthermore, epimedin C content increased as light intensity increased from L1 to L3, whereas epimedin C content decreased as light intensity increased from L3 to L5. Thus, L3 increased epimedin C by 483.6% (*p* < 0.05) compared with L1. Interestingly, icariin content was unaffected by light treatment. Icariin contents in plants under L4 and L1 were significantly higher than in plants under L5. Icariin contents under L2 and L3 were intermediate. Epimedin C was the major flavonoid in *Epimedium*.

### 2.5. Effect of Light Intensity on Leaf Dry Biomass and Medicinal-Ingredient Yield of Epimedium

**Figure 5:**
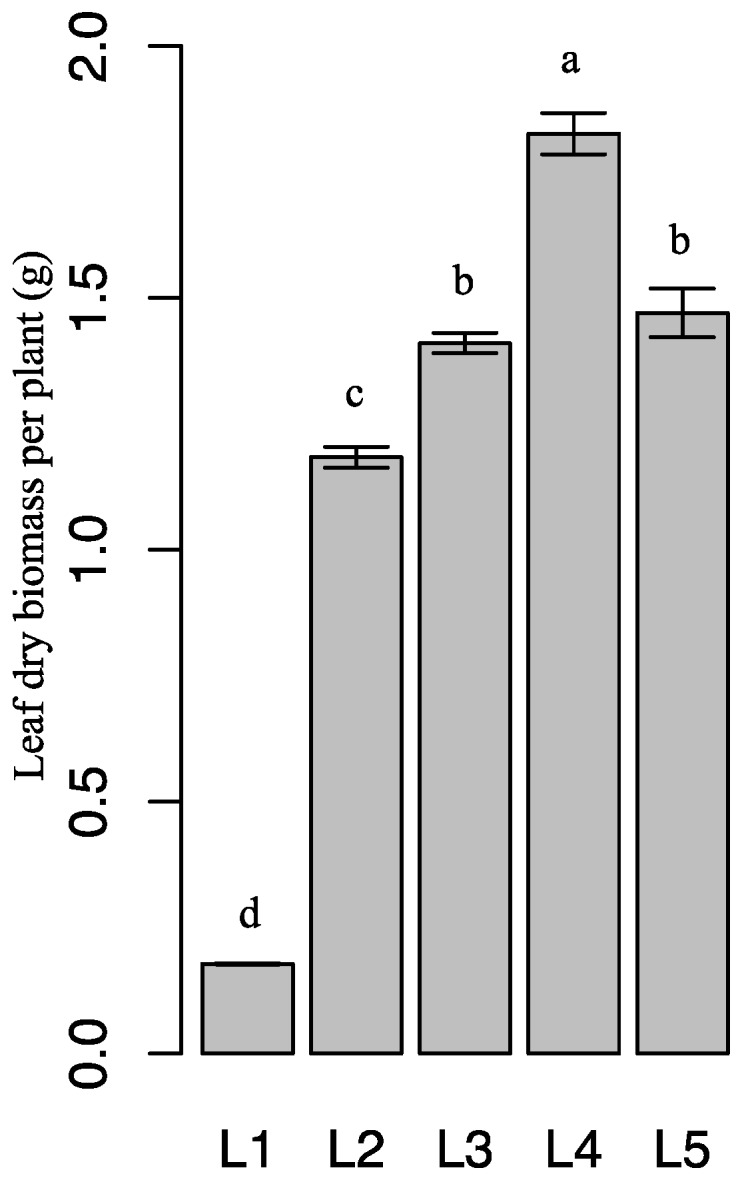
Leaf dry biomass of *E. pseudowushanense* under different light intensities. The data are expressed as mean ± SD. Different letters indicate significant differences between light intensity treatments (*p* < 0.05); *n* = 30.

Different light intensities were associated with differences in leaf dry biomass (Figure 5). The highest leaf dry biomass was observed in plants under L4. Leaf dry biomass gradually increased as light intensity increased from L1 to L4. L4 increased leaf dry biomass by 926.3% (*p* < 0.05) compared with L1. Interestingly, leaf dry biomass decreased as light intensity increased from L4 to L5.

**Figure 6:**
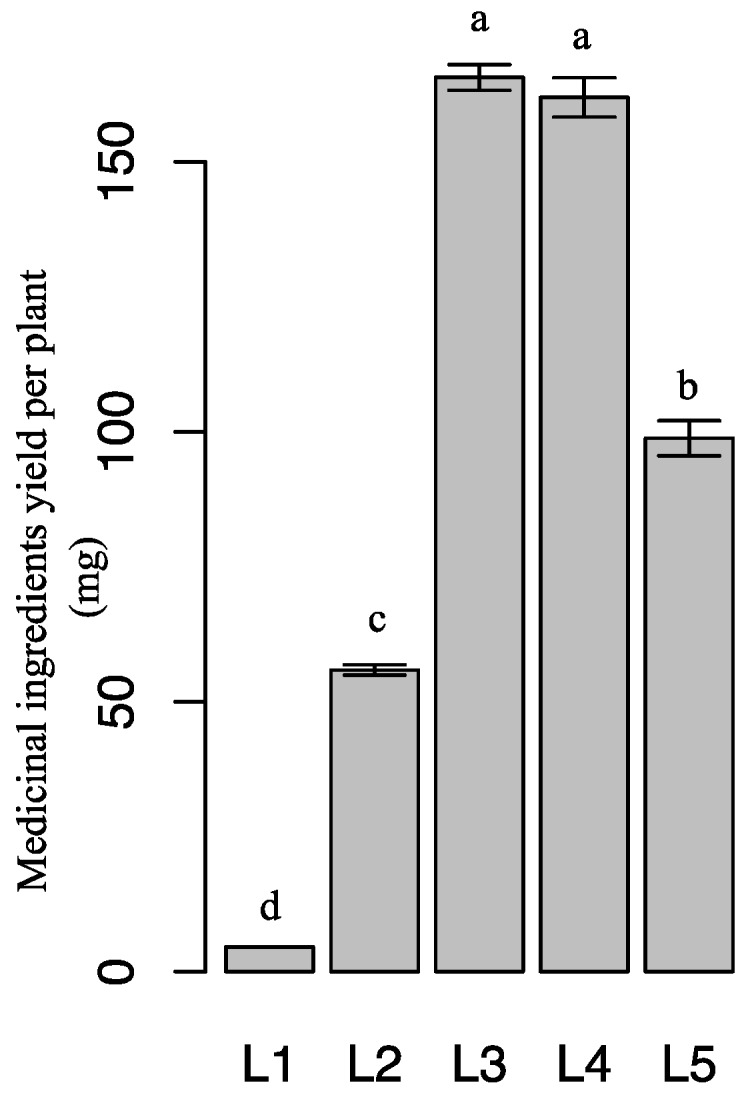
Medicinal-ingredient yields of *E. pseudowushanense* under different light intensities. The data are expressed as mean ± SD. Different letters indicate significant differences between light intensity treatments (*p* < 0.05); *n* = 30.

Given that the total of epimedin A, epimedin B, epimedin C, and icariin content in flavonoid glycosides was more than 90% in any treatment (data not shown), these four components represented flavonoid glycosides in *Epimedium*. Medicinal-ingredient yield was calculated as the product of flavonoid glycoside content and leaf dry biomass per plant. Figure 6 shows that plants under 60 days of L3 and L4 had higher medicinal-ingredient yields than under other treatments (*p* < 0.05). Medicinal-ingredient yield gradually increased as light intensity increased from L1 to L3, whereas it decreased by 40.3% (*p* < 0.05) as light intensity increased from L3 to L5.

## 3. Discussion

Although *Epimedium* is a shade-tolerant species, light intensity plays an important role in its survival, early growth, development of photosynthetic apparatus, and production of secondary metabolites [16,17,18]. Four major flavonoids including icariin, epimedin A, epimedin B, and epimedin C are quality indicators of *Epimedii Folium* [2]. Our study showed that medicinal-ingredient yields of individual plants were higher under 60 days of L3 and L4 treatment (Figure 6). This result suggests that the L3 and L4 are the optimal conditions for the medicinal ingredient production of *Epimedium*. Meanwhile, content of epimedin C, the major *Epimedium* flavonoid, was lower under low and high light intensity treatments, therefore reducing the medicinal-ingredient yield of individual plants under L1, L2, and L5 treatments (Figure 4). Interestingly, the production of different flavonoids was induced by different light ranges. A large number of other flavones were probably stimulated by lower light in addition to the four major components. In addition, epimedin A and epimedin B contents increased significantly as light intensity increased from L1 to L4, but decreased as light intensity increased from L4 to L5. Epimedin C content increased significantly as light intensity increased from L1 to L3, whereas it decreased as light intensity increased from L3 to L5. Furthermore, icariin content changed differently compared with other flavonoids. These results signify the complex relationships between light intensity and epimedin A, epimedin B, epimedin C, and icariin biosynthesis. Further studies are necessary to better understand the factors regulating the synthetic balance of the four medicinal flavones in the flavonoid pathway.

Leaf dry biomass influences the medicinal-ingredient yield of *Epimedium*. Leaf dry biomass is influenced by light intensity. Various plant characteristics, such as leaf area, number of branches, water content, and SPAD are influenced by light intensity [19]. Differences in plant morphology are documented in different species adapted to various light environments [20,21]. In the present study, *E. pseudowushanense* seedlings grown under relatively low light intensities (L1–L2) had larger leaves compared with those grown under high light intensities (L3–L5) (Figure 1). These findings are similar to the results reported by Tang and others [22]. Our study showed that *E. pseudowushanense* seedlings grown under L3–L5 treatments had significantly higher number of branches and lower water content (Figure 1). In *E. pseudowushanense*, the number of branches was directly associated with leaf biomass. More branches signify more leaves and more leaf dry biomass. Lower water content influenced leaf dry biomass similarly. Additionally, the highest Pn, gs, and T were observed in plants under L4 (Figure 2). Decreased Pn was associated with reduced T, gs, and Ci as light intensity increased from L4 to L5. This result indicates that stomatal limitation occurred and is presumably consistent with *E. pseudowushanense* stomatal traits. SPAD is another important factor determining photosynthetic rate and plant growth. In this study, we observed that SPAD decreased as light intensity increased from L3 to L5 (Figure 1), suggesting that excessive light intensity induced pigment damage [23]. This finding is consistent with the response of *T. hemsleyanum* [19]. Interestingly, higher SPAD and lower Pn were observed under L1 and L2. Thus, these results might be considered as responses to different light conditions [24].

Chloroplasts are the only organelles in which photosynthesis occurs. Photoreactions are localized in the internal chloroplast membrane (i.e., the thylakoid). Thylakoid structure and integrity are critical for effective photosynthesis [25]. In this study, leaves grown under L4 had better-developed grana and more thylakoids than those under other treatments (Figure 3), consistent with the highest photosynthetic rates and leaf dry biomass under L4. *E. pseudowushanense* chloroplasts were damaged under high light intensities (L5), thus decreasing flavonoid glycoside content because chloroplasts synthesize flavonoids in the plant cell [26,27]. Similar results were reported for *Camptotheca acuminata* seedlings [8].

*E. pseudowushanense* is most commonly utilized in the treatment of osteoporosis, liver cancer, and leukemia because of its high flavonoid content [4,28,29]. Based on previous studies on *Epimedium* species [30], the most suitable intensity for flavonoid accumulation in *E. sagittatum* ranged from 40–160 μmol·m^−2^·s^−1^. We set the L1 to L5 light regimes from 9.1–127.3 μmol·m^−2^·s^−1^. We conclude that L3 (54.6 ± 2.5 μmol·m^−2^·s^−1^) and L4 (90.9 ± 2.5 μmol·m^−2^·s^−1^) treatments achieved higher medicinal-ingredient yields after 60 days, and are therefore recommended for *Epimedium* cultivation. The present study provides a better understanding of the responses of growth, photosynthesis, and secondary metabolite accumulation in *E. pseudowushanense* seedlings exposed to various light intensities. Our results could serve as a theoretical basis for the standardized cultivation of *E. pseudowushanense*.

## 4. Materials and Methods

### 4.1. Plant Materials and Growth Conditions

**Figure 7:**
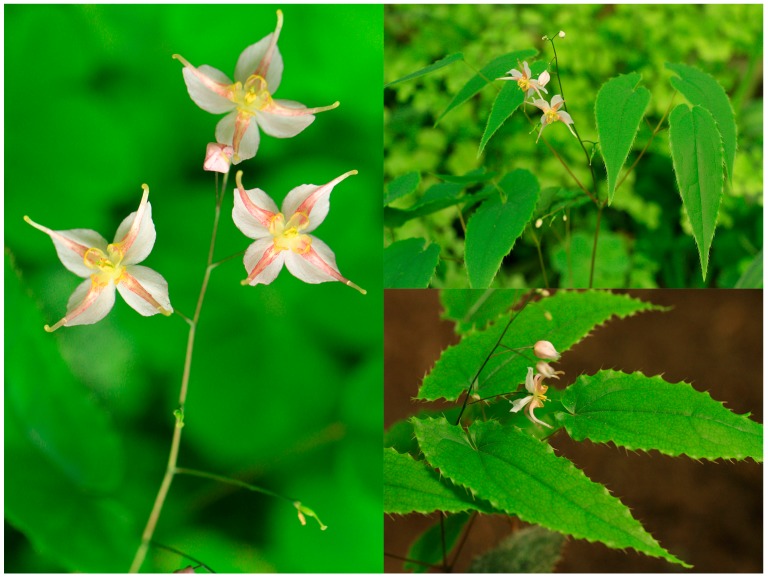
*E. pseudowushanense* flowers and leaves. There are two compound leaves on each stem and each compound leaf consisted of three leaflets.

*E. pseudowushanense* buds germinate in February. Flowering occurs from March to April and fruiting occurs in May [31]. *E. pseudowushanense* is cultivated under 10% to 30% irradiance in greenhouses. Its rhizome buds develop during winter. New leaves and stems with flowers develop in the following early spring. For this study, healthy, homogenous two-year-old *E. pseudowushanense* seedlings were collected from Lei Shan County (16° N, 108° E) in Guizhou Province. Each stem had two compound leaves. Each compound leaf had three leaflets (Figure 7). Seedlings were transferred to plastic pots (inner diameter: 10 cm, height: 10 cm) with drainage holes. One seedling was transferred per pot. All pots contained a substrate mixture of 75% peat and 25% vermiculite. On 26 December 2013, the transplanted seedlings were transferred to the greenhouse of the Institute of Medicinal Plant Development. Seedlings were then grown under shade. Each seedling was disinfected with 800-fold carbendazim solution. A completely randomized design with three replications per treatment and 10 seedlings per replicate was set up on 1 February 2014, or approximately one month after transferring. Inflorescences were removed upon blossoming to promote growth of the leaves utilized in medicinal treatments. Temperature range was set as 20–21 °C during the entire cultivation period. Humidity was maintained at 60%. The five light intensities were supplied by T5-fluorescent lamps at 16-h irradiation durations per day. Light treatment groups were as follows: L1 (9.1 ± 2.5 μmol·m^−2^·s^−1^), L2 (18.2 ± 2.5 μmol·m^−2^·s^−1^), L3 (54.6 ± 2.5 μmol·m^−2^·s^−1^), L4 (90.9 ± 2.5 μmol·m^−2^·s^−1^), and L5 (127.3 ± 2.5 μmol·m^−2^·s^−1^). Light intensities were measured by LI-6400 external quantum sensor (LI-COR, Lincoln, NE, USA) system. Sufficient water was provided for plants once every four days until the end of experimentation.

### 4.2. Plant Growth, Chlorophyll Content, and Leaf Dry Biomass

The number of newly germinated branches was recorded after 60 days of light treatment. Leaf areas of three leaflets were measured by LI-3000C (LI-COR, Lincoln, NE, USA). Fresh leaf weights were measured by an electronic balance. Leaf dry weights were measured after oven drying leaves at 60 °C to constant weight. Water content was calculated as: water content = 1 − dry weight/wet weight. Chlorophyll content was measured by a chlorophyll meter SPAD-502 (Konica Minolta Inc., Tokyo, Japan). Measurement light wavelengths were 650 nm and 940 nm. At the end of each treatment, all leaves of intact plants were collected to calculate leaf dry biomass.

### 4.3. Photosynthetic Parameters

Photosynthetic measurements were taken between 9:00 AM and 11:00 AM. Healthy, fully-developed, topmost leaflets of fronds were taken from three randomly selected plants per replicate treatment. Photosynthesis (Pn) was measured by a LI-6400 portable photosynthesis system (Li-Cor, Inc., Lincoln, NE, USA) with a standard leaf chamber equipped with a 6400-02B LED light source. Data were recorded under 21% O_2_, 500 μmol (CO_2_)·m^−2^·s^−1^ air concentration, 60% relative humidity, 200 μmol (CO_2_)·s^−1^ air flow, and 20 ± 0.5 °C temperature. Vapor pressure deficit (VPD) was calculated with leaf and air temperatures and relative humidity. VPD was similar among the five experimental treatments.

### 4.4. Assessment of Chloroplast Ultrastructure

To observe the chloroplast ultrastructure of mesophyll cells in *E. pseudowushanense* seedlings subjected to different light intensities for 60 days, a fully expanded leaflet from a randomly selected plant from each replicate per treatment was collected. Fresh leaves were cut into 4–6 slices (1 mm^2^) at their adaxial surfaces for transmission electron microscopy. Sections were placed immediately in 2.5% (*v*/*v*) glutaraldehyde (0.1 M phosphate buffer, pH 7.2), then de-gassed and fixed for at least 4 h. Samples were then post-fixed with 1% (*v*/*v*) osmium acid. After fixation, samples were washed three times with the same buffer for 15 min. Specimens were dehydrated by a graded ethanol series (50%, 70%, 90%, 95%, and 100% at 30 min each) then embedded in epoxy resin, from which ultrathin sections were prepared. The ultrathin sections were sequentially stained with uranyl acetate and lead citrate and then examined with a transmission electron microscope (JEM-1400, Hitachi, Tokyo, Japan).

### 4.5. Flavonoid Content and Medicinal-Ingredient Yield

One ternate leaf was collected per plant after 30, 60, 90, and 120 days of exposure to different light intensity treatments. Fresh leaves of 10 seedlings per replicate were fixed for 3 min in a microwave, then dried to a constant weight at 60 °C. Sample pretreatment was performed according to the Chinese Pharmacopoeia Commission (2010). Samples were crushed and sifted through a No. 3 pharmacopoeia sieve. Approximately 200 mg of sample was added to 50 mL 70% ethanol then extracted by ultrasonication for 30 min. The extract was then filtered with a 0.45 μm microfiltration membrane for HPLC analysis. Eluent A contained double distilled water and eluent B contained acetonitrile. The gradient elution program was as follows: 0–17 min (25%–26% B), 17–26 min (26%–100% B). The column was washed with 100% eluent B between every two samples for 15 min and then re-equilibrated with 25% eluent B for 10 min. Elution conditions were as follows: 1.0 mL·min^−1^ flow rate, 25 °C column temperature, 270 nm detection wavelength, and 20 μL injection volume. Six gradient concentrations of epimedin A (3.1–32.3 μg·mL^−1^), epimedin B (3.1–32.3 μg·mL^−1^), epimedin C (20.5–520.6 μg·mL^−1^), and icariin (3.1–32.3 μg·mL^−1^) were prepared. A Zorbax SB-C18 column (Agilent Technologies, Palo Alto, CA, USA) was utilized as the chromatographic column in chromatographic analysis (250 mm × 4.6 mm I.D., 5 μm). HPLC analysis data were processed by PerkinElmer ChemStation software (version 6.3.1, PerkinElmer, Waltham, MA, USA).

Medicinal ingredient yield was calculated as: medicinal-ingredient yield = (epimedin A content + epimedin B content + epimedin C content + icariin content) × leaf dry biomass.

### 4.6. Data Analysis

Statistical analysis was conducted with one-way ANOVA software (R language version 3.1.1, https://www.r-project.org/about.html). Differences between means were detected with Tukey HSD test. *p* value was set at 0.05 and 0.01 for ANOVA and Tukey HSD tests, respectively.

## Figures and Tables

**Table 1:** Calibration data of four analytes.

Analyte ^a^	Linear Range (μg·mL^−1^)	Calibration Equation ^b^	r^2^	LOD (μg·mL^−1^)	LOQ (μg·mL^−1^)	Intra-Day RSD (%) (*n* = 6)			Inter-Day RSD (%) (*n* = 6)			Recovery and RSD (%) (Mean, *n* = 6)
						L1	L3	L5	L1	L3	L5	
1	3.1~32.2	*Y* = 7701.546*x* + 9983	0.9992	0.26	0.81	2.46	1.91	1.36	1.06	0.98	1.39	105.54, 3.32
2	3.1~32.2	*Y* = 5548.525*x* + 15,762	0.9995	0.29	0.95	3.27	2.38	1.57	1.62	1.78	1.20	104.06, 2.96
3	20.5~520.6	*Y* = 7171.274*x* + 31,544	0.9993	0.03	0.11	3.36	1.50	2.02	1.83	1.19	1.14	102.95, 3.37
4	3.1~32.2	*Y* = 9108.011*x* + 6141	0.9996	0.11	0.37	2.50	1.66	1.46	1.79	1.54	1.44	104.92, 2.70

^a^ The compound codes 1, 2, 3, and 4 of each analyte refers to epimedin A, epimedin B, epimedin C, and icariin, respectively; ^b^ Y: peak area; X: concentration of compound (μg·mL^−1^).

## Abbreviations

Pn	Net photosynthetic rate
gs	Stomatal conductance
SPAD	Soil and Plant Analyzer Development (the relative content of chlorophyll)
T	Transpiration rate
WUE	Water use efficiency
Ci	Intercellular CO_2_ concentration
SG	Starch grains
GL	Grana lamellae
VPD	Vapor pressure deficit
